# Is it time for Heart–Brain clinics? A clinical survey and proposition to improve current care for cognitive problems in heart failure

**DOI:** 10.1002/clc.24200

**Published:** 2024-01-06

**Authors:** Charlotte M. Nijskens, Elias G. Thomas, Hanneke F. M. Rhodius‐Meester, Mat J. A. P. Daemen, Geert Jan Biessels, M. Louis Handoko, Majon Muller

**Affiliations:** ^1^ Department of Internal Medicine, Geriatrics Section Amsterdam UMC Location Vrije Universiteit Amsterdam Amsterdam The Netherlands; ^2^ Amsterdam Public Health Amsterdam UMC Amsterdam The Netherlands; ^3^ Department of Neurology, Alzheimer Center Amsterdam Amsterdam UMC Location Vrije Universiteit Amsterdam Amsterdam The Netherlands; ^4^ Department of Geriatric Medicine Oslo University Hospital Oslo Norway; ^5^ Amsterdam Neuroscience Amsterdam UMC Amsterdam The Netherlands; ^6^ Department of Pathology Amsterdam UMC Location University of Amsterdam Amsterdam The Netherlands; ^7^ Amsterdam Cardiovascular Sciences Amsterdam UMC Amsterdam The Netherlands; ^8^ Department of Neurology, UMC Utrecht Brain Center University Medical Center Utrecht The Netherlands; ^9^ Department of Cardiology Amsterdam UMC Location Vrije Universiteit Amsterdam Amsterdam The Netherlands

**Keywords:** cardiovascular dysfunction, cognitive decline, heart failure, vascular cognitive impairment, vascular dementia

## Abstract

**Background:**

Cognitive impairment is highly prevalent among patients with heart failure (HF). International guidelines on the management of HF recommend screening for cognitive impairment and tailored care for patients with cognitive impairment. However, practical guidance is lacking. In this study, we explore cardiologists' perspective on screening and care for cognitive impairment in patients with HF. We give an example of a multidisciplinary Heart–Brain care pathway that facilitates screening for cognitive impairment in patients with HF.

**Methods:**

We distributed an online survey to cardiologists from the Dutch working groups on Geriatric Cardiology and Heart Failure. It covered questions about current clinical practice, impact of cognitive impairment on clinical decision‐making, and their knowledge and skills to recognize cognitive impairment.

**Results:**

Thirty‐six out of 55 invited cardiologists responded. Only 3% performed structured cognitive screening, while 83% stated that not enough attention is paid to cognitive impairment. More than half of the cardiologists desired more training in recognizing cognitive impairment and three‐quarters indicated that knowing about cognitive impairment would change their treatment plan. Eighty percent agreed that systematic cognitive screening would benefit their patients and 74% wished to implement a Heart–Brain clinic. Time and expertise were addressed as the major barriers to screening for cognitive impairment.

**Conclusion:**

Although cardiologists are aware of the clinical relevance of screening for cognitive impairment in cardiology patients, such clinical conduct is not yet commonly practiced due to lack of time and expertise. The Heart–Brain care pathway could facilitate this screening, thus improving personalized care in cardiology.

## 1 INTRODUCTION

As the age of the global population increases and survival after a major acute cardiovascular event improves, chronic cardiovascular diseases, such as heart failure (HF), are on the rise.^1^ In patients with HF, cognitive problems—including mild cognitive impairment and dementia—are common,^2^, ^3^, ^4^ with a prevalence ranging from 20% to 40% in these patients.^5^, ^6^ In these patients, the most commonly affected cognitive domains are memory, attention, problem solving, and processing speed.^4^ Both HF and cognitive impairment impose a high burden on patients, caregivers, and society, especially when existing concomitantly. For example, HF patients with cognitive impairment have a doubled readmission rate, faster functional decline and a fivefold increase in premature mortality compared to cardiac patients with normal cognitive function.^7^, ^8^ In addition, self‐care and adherence to (often complex) therapy for HF is poorer in these patients.^9^, ^10^

To improve care for patients with HF, cardiologists should be aware of the concomitance of HF and cognitive impairment and structural cognitive screening should take place. In practice, cognitive impairment is often not recognized by the treating physician.^11^ During a short consultation, clinicians may fail to notice signs of cognitive impairment, like impaired problem solving and decreased processing speed, since they can be less obvious than memory loss.^12^ In current HF guidelines, cognitive impairment is addressed as a risk factor for poorer self‐care and significant adjustments in HF treatment plans is recommended in the event of dementia.^13^, ^14^ However, these guidelines lack practical instructions on which patients need to be screened and the timing of screening, which hinders the provision of tailored care. Although the American Heart Association gives some examples of tools for cognitive screening, they do not recommend how and when to use these tools.^13^ This gap in clinical recommendations most likely leads to cases of significant cognitive impairment going unnoticed in current clinical practice.

**Figure 1:**
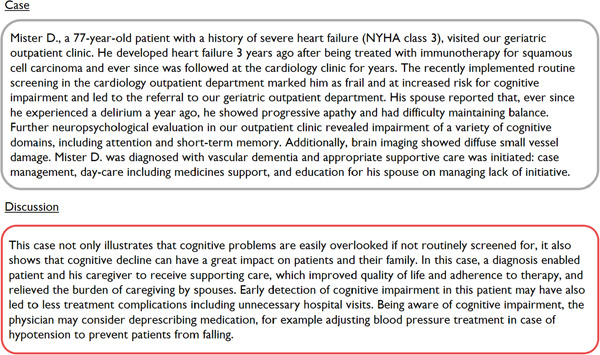
Case of a patient with heart failure that illustrates the outcome of routine screening for cognitive impairment.

In the Amsterdam UMC, we recently initiated the implementation of routine screening for cognitive impairment at the Heart–Brain clinic from the Heart–Brain Consortium.^15^, ^16^ In this clinic, nurses, cardiologists, and (internist‐)geriatricians perform screening and improve management of cognitive impairment in patients with HF in a care pathway. In Figure 1, we illustrate this care pathway by a patient case that demonstrates how screening for cognitive impairment was performed and how it improved care. To investigate whether cardiologists support a “heart‐brain care pathway” and to further adjust this care pathway to cardiologists' needs and wishes, more insight is needed into cardiologists' perspectives on cognitive impairment in this patient group. In this article, we explore the perspectives of cardiologists on cognitive impairment in their patient population using a national, clinical survey. In addition, we present our care pathway, which may aid in the recognition of cognitive impairment in current care for chronic cardiac patients.

## 2 METHODS OF THE CLINICAL SURVEY

A survey among cardiologists was performed using a standardized online questionnaire to assess their views on the impact of cognitive impairment in their patients. This survey entailed 20 multiple‐choice and two open‐ended questions and was developed by E.G.T., after which input was gathered from a geriatrician (H.F.M.R.), an internist‐geriatrician (M.M.), and a cardiologist (M.L.H.). Questions were asked about current clinical practice, cardiologists' view on impact of cognitive impairment on clinical decision‐making, and their role and ability to recognize these impairments. The open‐link questionnaire was sent to cardiologists from the Dutch working group on Geriatric Cardiology and the Dutch working group on HF, and they were asked to distribute the survey to their colleagues. The complete survey can be found in Supporting Information S1: Appendix A.

## 3 RESULTS OF THE CLINICAL SURVEY

Thirty‐six out of 55 invited cardiologists filled out the questionnaire. The mean age of participants was 48 years, and two‐thirds of the participants were male. More than half of the participants—62%—had more than 10 years of experience in the treatment of HF.

Cardiologists generally underestimated the prevalence of cognitive impairment; only one in four cardiologists estimated the correct prevalence of 20%–40%. One in three cardiologists regularly referred their patients to the geriatrician or neurologist for cognitive analysis. Although four out of five physicians stated that they did not know which cognitive problems to expect in their patients, 70% cooperated with geriatricians or primary care physicians to improve their knowledge of cognitive impairment. Eighty‐three percent disagreed with the statement that enough attention is paid to cognitive problems in current cardiology practice. The major barrier to improving this shortcoming was time. In general, a diagnosis of cognitive impairment was considered relevant for clinical practice, and three out of four cardiologists would adjust their treatment plan when noticing cognitive impairment. Adjustments would include more restraint in performing invasive treatments, paying more attention to side effects of cardiac medication, and accepting a higher blood pressure. Although cardiologists were aware of the importance of cognitive impairment, the number of them performing standardized screening was low (3%). Most cardiologists were convinced that standardized screening tools were more appropriate to detect cognitive problems than their subjective evaluation. Moreover, 80% believed that systematic cognitive screening would benefit health care for cardiac patients. However, only one cardiologist used screening tools.

Possible contributing factors to this discrepancy were also assessed in our survey: more than half of the respondents (53%) indicated not being educated on cognitive problems in patients with HF or chronic cardiac conditions during their specialty training. Only few cardiologists (8%) indicated that recognizing cognitive impairment in cardiac patients was their responsibility, while the majority considered it as the responsibility of geriatricians (14%) or primary care physicians (47%). One in four participants indicated that it is the shared responsibility of all physicians treating the patient. Most participants—77%—did not consider it feasible to pay more attention to cognition within cardiac care, indicating a desire for a clear protocol besides the aforementioned experience of having too little knowledge about cognition. Most cardiologists wanted more training in recognizing cognitive complaints in their patients (65%) and tailoring care for these patients (71%). When asked if they would implement a specialized outpatient clinic for these patients in their hospital, 75% stated they would.

## 4 DISCUSSION

### 4.1 Interpretation of findings

The most important findings from this survey show that most cardiologists acknowledge the importance of recognizing cognitive impairment in their patients and desire more guidance for screening. Most cardiologists in this survey would be more reluctant to start complex drug regimens and invasive procedures when treating a patient with cognitive impairment. Yet, three in four cardiologists underestimated the prevalence of cognitive impairment in their patients. The main barriers to performing cognitive screening were lack of time and knowledge, as well as lack of sense of responsibility and feasibility. These findings underline the need for multidisciplinary clinics where systematic cognitive assessment in a manner focuses on the connection between heart and brain.

In line with recommendations in the European and American guidelines on HF, we found that cardiologists recognize the importance of early detection of cognitive impairment. These guidelines state that older patients with HF are at increased risk for cognitive decline and that treating physicians should be aware of cognitive impairment in their patients.^13^ The prevalence of cognitive impairment was underestimated by most cardiologists in this survey. Cardiologists also stated that they did not feel competent to perform cognitive screening for cognitive impairment. We suspect that this lack of confidence will pose an even greater obstacle to cardiologists without special interest in geriatric cardiology. It is not surprising that cardiologists do not feel confident to perform cognitive screening, since physicians, in general, are not sufficiently trained to recognize cognitive problems in their patients.^17^ A previous study showed that the number of cognitive problems perceived by physicians through clinical impression significantly lags behind the number of objectively assessed cognitive impairments.^18^ In the event of vascular cognitive impairment, this difference may be due to symptoms like slowed thinking and apathy, which can be less evident to clinicians than typical hallmarks of Alzheimer disease like memory loss.^12^

The main motivations for cardiologists to screen for cognitive impairment were refraining from invasive procedures, modifying blood pressure management, and closer monitoring of adverse drug events. In accordance with the international HF guidelines, early detection of cognitive impairment and comorbid conditions in patients with HF should lead to tailored care, which may reduce the impact of HF on cognitive function and potentially prevent further brain damage and decline in cognitive function.^19^, ^20^, ^21^ Tailored cardiac care could also enable deprescribing harmful medication. Although there is no clear evidence for different treatment goals in older HF patients with cognitive impairment, less strict targets are fitting in cases of severe cognitive impairment or reduced life expectancy.^22^ At the same time, clinical benefits may be derived from more frequent blood pressure measurements to prevent hypotension, as well as measures to prevent falling.^23^ Another important reason why cardiologists chose to screen for cognitive impairment was the enhancement of treatment compliance. Patients with cognitive impairment often find it problematic to self‐manage the medication and dietary restrictions required for HF treatment. Simplified treatment regimens or supportive home care could enable compliance and potentially prevent hospitalization for acute HF decompensation.^24^ Furthermore, although it was not addressed in this survey, discussing advance care planning is especially relevant in this patient group.^25^

Since lack of time and knowledge were the main reasons for the omission of cognitive screening by cardiologists, structured screening in cardiology clinics should be instituted to adequately recognize cognitive problems. Once cognitive problems are observed, patients should be referred to a multidisciplinary clinic where systematic cognitive assessment can be performed, while focusing on the connection between heart and brain. The results of this study indicated that cardiologists would welcome the implementation of such clinics in their hospital.

### 4.2 Introduction of Heart–Brain clinic

**Figure 2:**
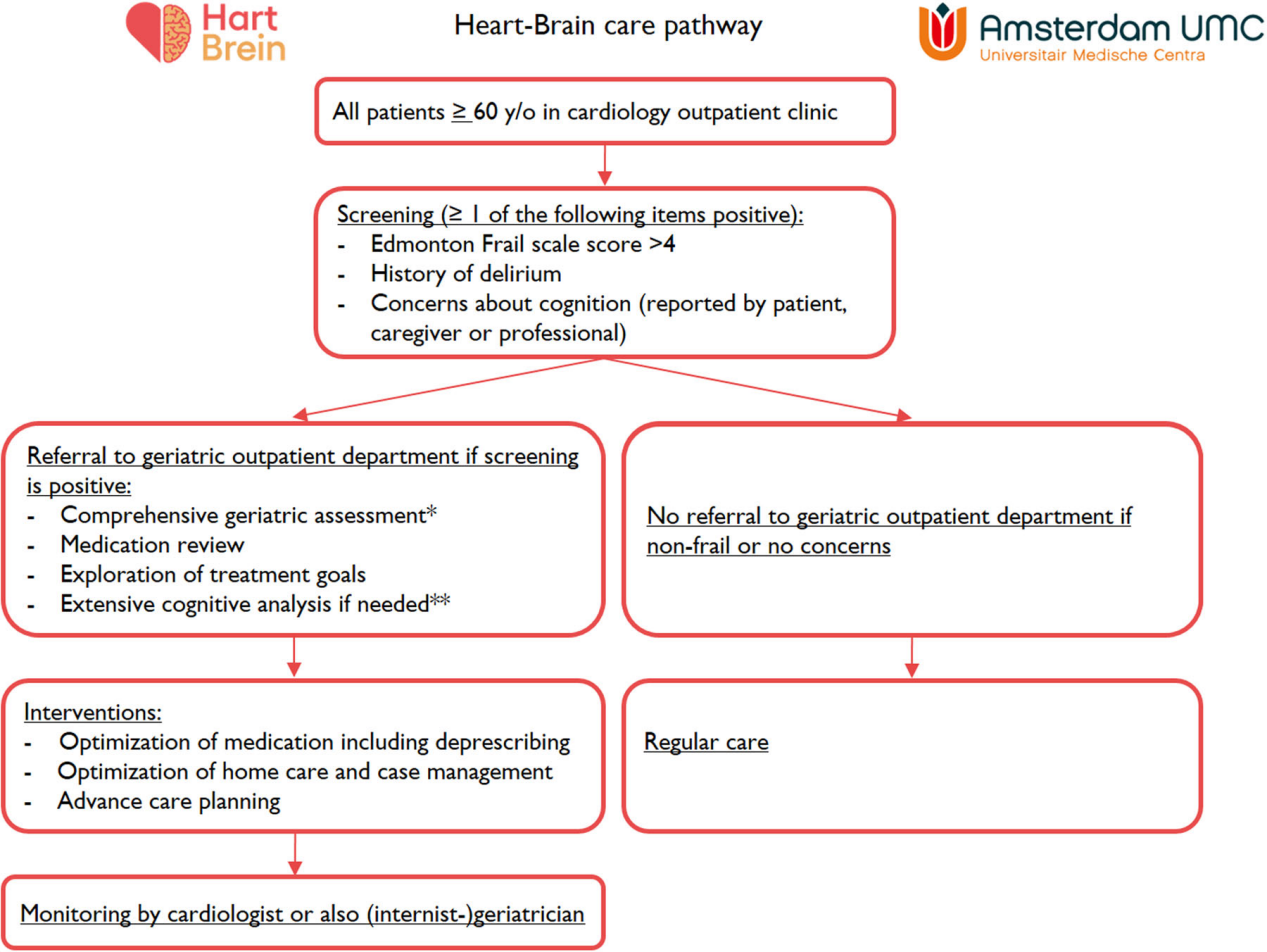
Screening and intervention strategy in the Heart–Brain clinic of Amsterdam UMC. * Comprehensive geriatric assessment: this assessment includes muscle strength (handgrip strength, gait speed), Mini Nutritional Assessment—Short From (MNA‐SF),^26^ *Mini‐Mental State Examination (MMSE)*,^27^ *Montreal Cognitive Assessment (MoCA)*,^28^ *and the Geriatric Depression Scale*.^29^ ** Extensive cognitive analysis is performed in those with suspected cognitive impairment. It includes neuropsychological examination and brain MRI. Results are discussed and diagnoses are made in a multidisciplinary consensus meeting.

We recommend a stepwise screening program for cognitive impairment in all patients with HF. In Figure 2, we illustrate our multidisciplinary Heart–Brain care pathway, which is part of the first Heart–Brain clinic opened in 2022 in the Amsterdam UMC. All patients at this cardiology outpatient clinic aged 60 years or older are screened for a history of delirium, cognitive complaints, and frailty with the Edmonton Frail Scale, which includes a quick cognitive test (clock drawing).^30^ Nurses are trained for 1 hour to perform this screening. Once this screening is incorporated in clinical routine, it takes only 5 minute to perform during one of the earlier outpatient visits. When patients are considered to be frail and at increased risk for cognitive impairment, a step‐wise program is provided including geriatric assessment, medication review, and exploration of treatment goals. This geriatric assessment is performed by a trained nurse according to a standardized work‐up and includes muscle strength (handgrip strength, gait speed), Mini Nutritional Assessment—Short From,^26^ Mini‐Mental State Examination,^27^ Montreal Cognitive Assessment,^28^ and the Geriatric Depression Scale.^29^ In the event of suspected cognitive impairment, an extensive cognitive analysis is performed, including neuropsychological examination and brain MRI. Results are discussed and diagnoses are made in a multidisciplinary consensus meeting. Subsequently, attention is paid to personalized medical treatment (including deprescribing), supportive care, optimization of home care, and advance care planning to improve quality of life, ensure adherence to therapies and reduce care consumption and related costs. The findings and interventions are reported to the cardiologist and general practitioner. Being aware of the outcomes of the current survey, we are planning to actively inform the cardiologists and/or specialized HF nurse (e.g., through multidisciplinary meetings) to educate them on cognitive impairment and assist in advance care planning in the event of dementia. Depending on the diagnosis, patients remain under the care of the cardiologist or of the (internist‐)geriatrician as well.

### 4.3 Limitations of this study

The main limitation of this study is the study population. Only cardiologists from the working groups on geriatric cardiology and working groups on HF took part in our survey. These physicians are probably more aware of cognitive impairment in patients with HF than (general) cardiologists who mainly treat younger cardiac patients, whose cognitive functioning is normally intact. We therefore may have overestimated cardiologists' knowledge about cognitive screening and the implications for HF treatment. However, this possibility emphasizes the need for multidisciplinary Heart–Brain clinics even further. In these clinics, structural screening takes place even without the involvement of a cardiologist who might be unaware of cognitive impairment in his patients.

### 4.4 Future directions

With the results of the survey and an example of a multidisciplinary care pathway, we hope to inspire physicians and nurses involved in the care for older HF patients to implement this stepwise screening for cognitive impairment. We will soon evaluate this care pathway and report on its challenges and successes. Independent of these results, we recommend that future guidelines incorporate more recommendations for practical guidance on how to screen and improve the care for patients with cognitive impairment and HF. Also, improvement in education on cognitive impairment in cardiology will facilitate the empowerment of cardiologists to implement tailored care for their patients with cognitive impairment.

## CONFLICTS OF INTEREST STATEMENT

H.F.M.R. performs contract research for Combinostics; all funding is paid to her institution. M.L.H. received educational/speaker/consultancy fees from Novartis, Boehringer Ingelheim, Daiichi Sankyo, Vifor Pharma, AstraZeneca, Bayer, MSD, Quin and Abbott; all not related to this work. The remaining authors declare no conflict of interest.

## Supporting information

Supporting information.

## Data Availability

The data underlying this article will be shared on reasonable request to the corresponding author.
