# Supplementary material for: Is it time for Heart–Brain clinics? A clinical survey and proposition to improve current care for cognitive problems in heart failure
Source: Clin Cardiol. 2024 Jan 6;47(1):e24200. doi: 10.1002/clc.24200 (PMC10785189; doi:10.1002/clc.24200)
Supplement: Supplementary file 1 — Supporting information. [file CLC-47-e24200-s001.docx]

**Appendix A**

This Dutch survey questionnaire was sent to members of a group of cardiologists treating HF patients. Potential respondents received an email describing the goal of the survey and were asked to participate anonymously using a non-personal link in the email to the online questionnaire. Three weeks after the initial approach, a reminder was sent.

The online questionnaire consisted of 22 questions, mostly in multiple-choice form, using a Likert scale to rate agreement or disagreement with the provided statements. During and at the end of the questionnaire, the cardiologists were given the opportunity to provide additional comments.

Clinical questionnaire as presented to the participants, including results. Between brackets, the percentage of participants who selected the concerned answer is presented.

1. What is your age? (respondents: 36)

*Mean age: 48. Range: 24 – 66.*

1. What is your gender? (respondents: 36)
   1. Male [67%]
   2. Female [33%]
   3. Other [0%]
   4. I don’t want to disclose my gender [0%]
2. In what type of hospital do you work? (respondents: 36)
   1. University hospital [50%]
   2. Large teaching hospital [36%]
   3. Community hospital [14%]
   4. Private institution [3%]
3. How many years of experience do you have in treating patients with heart failure? (respondents: 36)
   1. 1-10 years [39%]
   2. 10-20 years [25%]
   3. 20-30 years [22%]
   4. 30-40 years [14%]
4. How many patients with heart failure have cognitive problems (please estimate)? (respondents: 36)
   1. 0-20% [25%]
   2. 20-40% [50%]
   3. 40-60% [25%]
   4. More than 60% [0%]
5. I am familiar with the problems in cognition that are prevalent in patients with heart failure. (respondents: 36)
   1. Strongly agree [3%]
   2. Agree [22%]
   3. Neutral [42%]
   4. Disagree [28%]
   5. Strongly disagree [3%]
6. As a part of my clinical training, I have been taught how to recognize cognitive problems in my patient population. (respondents: 36)
   1. Strongly agree [0%]
   2. Agree [11%]
   3. Neutral [11%]
   4. Disagree [52%]
   5. Strongly disagree [22%]
7. Assessment of cognitive problems is primarily the responsibility of: (respondents: 36)
   1. The cardiologist [8%]
   2. Specialist cardiology nurses [6%]
   3. Primary care doctors [47%]
   4. Geriatricians [14%]
   5. Other, […] [25%]
      *Answers included: multi-disciplinary effect, joined effort, all of the above*
8. I frequently refer patients with heart failure to the neurologist or geriatrician for cognitive evaluation. (respondents: 36)
   1. Strongly agree [3%]
   2. Agree [31%]
   3. Neutral [25%]
   4. Disagree [25%]
   5. Strongly disagree [11%]
9. I use systematic tools or screening tools to assess for cognitive function. (respondents: 36)
   1. Strongly agree [0%]
   2. Agree [3%]
   3. Neutral [11%]
   4. Disagree [39%]
   5. Strongly disagree [47%]
10. I feel that my clinical judgement is more suited to assess risk for cognitive problems than formal tools. (respondents: 36)
    1. Strongly agree [0%]
    2. Agree [14%]
    3. Neutral [19%]
    4. Disagree [58%]
    5. Strongly disagree [6%]
11. If a patient has cognitive problems, this influences my cardiac treatment plan. (respondents: 36)
    1. Strongly agree [8%]
    2. Agree [67%]
    3. Neutral [6%]
    4. Disagree [14%]
    5. Strongly disagree [6%]
       Free text answers included: *earlier referral to geriatrician, more lenient blood pressure management, less invasive treatment, assessment of therapy compliance*
12. Within current care, enough attention is paid to cognitive problems in patients with chronic cardiac conditions. (respondents: 36)
    1. Strongly agree [0%]
    2. Agree [0%]
    3. Neutral [17%]
    4. Disagree [69%]
    5. Strongly disagree [14%]
13. In your opinion, what are elements of care which are currently missing for heart failure patients with cognitive problems? (respondents: 36)

*Free text answers included: systematic assessment, time, knowledge amongst cardiologists , recognition of cognitive problems*

1. Do you currently work together with other disciplines to provide care for patients with heart failure and cognitive problems? (respondents: 36)
   1. No [28%]
   2. Yes, with geriatricians [61%]
   3. Yes, with family doctors [19%]
   4. Yes, with neurologists [6%]
2. What amount of time would be acceptable for you to spend on screening to assess cognitive function? (respondents: 34)
   1. Less than one minute [24%]
   2. 1-2 minutes [26%]
   3. 2-5 minutes [35%]
   4. More than 5 minutes [15%]
3. Within current clinical care, it is feasible to pay more attention to cognitive problems in cardiology. (respondents: 34)
   1. Strongly agree [0%]
   2. Agree [9%]
   3. Neutral [12%]
   4. Disagree [56%]
   5. Strongly disagree [21%]
4. A routine screening tool for cognitive problems would enhance adequate treatment for cardiology patients. (respondents: 34)
   1. Strongly agree [15%]
   2. Agree [65%]
   3. Neutral [12%]
   4. Disagree [9%]
   5. Strongly disagree [0%]
5. If patients are referred to the geriatrician for assessment of their cognitive function, to which extent would you like to be involved in this process? (respondents: 34)
   1. Not at all, my patients will give me the relevant information if needed [0%]
   2. I will read the report in the patient file [62%]
   3. I would like to be [or have a cardiology staff member be] present in a consensus meeting including the geriatrician [29%]
   4. I would like to get informed via an online platform [0%]
   5. Other [9%]

*Free text answers included: specialized nurses should attend a multidisciplinary meeting, there should be a joined outpatient consultation by the cardiologist and geriatrician*

1. I would like to receive more training on recognizing cognitive problems in cardiac patients. (respondents: 34)
   1. Strongly agree [9%]
   2. Agree [56%]
   3. Neutral [26%]
   4. Disagree [9%]
   5. Strongly disagree [0%]
2. I would like to receive more training adequately managing cardiac patients with comorbid cognitive problems. (respondents: 34)
   1. Strongly agree [6%]
   2. Agree [65%]
   3. Neutral [21%]
   4. Disagree [9%]
   5. Strongly disagree [0%]
3. Is this something you would be interested in implementing in your own hospital? (respondents: 34)
   1. Yes [74%]
   2. No [26%]
